# Potential Therapeutic Targeting Neurotransmitter Receptors in Diabetes

**DOI:** 10.3389/fendo.2022.884549

**Published:** 2022-05-20

**Authors:** Xiaohui Pan, Shibing Tao, Nanwei Tong

**Affiliations:** ^1^Department of Endocrinology and Metabolism, West China Hospital, Sichuan University, Chengdu, China; ^2^Laboratory of Diabetes and Islet Transplantation, Center for Diabetes and Metabolism Research, West China Hospital, Sichuan University, Chengdu, China; ^3^Department of Endocrinology, Ziyang First People’s Hospital, Ziyang, China

**Keywords:** glucose homeostasis, neurotransmitters, neurotransmitter receptor, pancreatic islets, type 2 diabetes

## Abstract

Neurotransmitters are signaling molecules secreted by neurons to coordinate communication and proper function among different sections in the central neural system (CNS) by binding with different receptors. Some neurotransmitters as well as their receptors are found in pancreatic islets and are involved in the regulation of glucose homeostasis. Neurotransmitters can act with their receptors in pancreatic islets to stimulate or inhibit the secretion of insulin (β cell), glucagon (α cell) or somatostatin (δ cell). Neurotransmitter receptors are either G-protein coupled receptors or ligand-gated channels, their effects on blood glucose are mainly decided by the number and location of them in islets. Dysfunction of neurotransmitters receptors in islets is involved in the development of β cell dysfunction and type 2 diabetes (T2D).Therapies targeting different transmitter systems have great potential in the prevention and treatment of T2D and other metabolic diseases.

## Introduction

Glucose homeostasis is critical for life maintenance, and the normal glucose ranges for the body are set by the biological defended level of glycemia (BDG_L_) in the central nervous system (CNS). The neuronal populations in the arcuate nucleus of the hypothalamus are crucial for the regulation of energy balance, primarily in the control of food intake (i.e., appetite), which makes the CNS an indispensable part of metabolism (1). The pancreatic islets are key parts of glucose homeostasis. Pancreatic β cells are the only source of the glucose-lowering hormone insulin in the body. Dysfunction of β cells leads to impaired or insufficient insulin secretion, which results in hyperglycemia and diabetes. Nutrients in circulation, such as glucose, fatty acids and amino acids, can act on islets directly or indirectly to modulate the secretion of hormones from islets to regulate blood glucose.

Glucose is the most important insulin secretagogue. When blood glucose is higher than the Km of glucose transporters on the β cell membrane, they work to take up glucose into cells. The metabolism of glucose generates adenosine triphosphate (ATP), resulting in closure of ATP-sensitive K^+^ channels (K_ATP_) to trigger membrane depolarization, electrical activity and opening of voltage-dependent Ca^2+^ channels (VDCCs), increasing the intracellular Ca^2+^concentration ([Ca^2+^]_i_) and initiating exocytosis of insulin granules (2, 3). The other cells in the islets share a similar secretion pattern and play roles in the maintenance of glucose homeostasis. The α cells of the pancreatic islets secrete glucagon to raise blood glucose in response to hypoglycemia, and the δ cells secrete somatostatin to inhibit the secretion of both insulin and glucagon. Appropriate communication within the islets as well as between islets and other organs is needed to maintain glucose homeostasis under different situations (4).

Neurotransmitters are a group of signaling molecules secreted by neurons that modulate the function of the nervous system, including amino acids, monoamines, peptides, and purines (5). The blood–brain barrier (BBB) restricts the communication of neurotransmitters between the CNS and periphery to keep the brain functioning correctly. The islet is a mini-organ vascularized and innervated substantially, and CNS-derived neurotransmitters can function on islets through sympathetic and parasympathetic nerves (6, 7). Moreover, neurotransmitters in the CNS can also be synthesized in the periphery, including islets, and regulate insulin secretion in a glucose-dependent or glucose-independent manner to maintain glucose homeostasis. Upon binding with corresponding receptors on the membrane, the signaling molecules exert stimulatory or inhibitory effects on hormones secretion. There are two major types of neurotransmitter receptors: inotropic receptors and metabotropic receptors (8). Ionotropic receptors provide ligand-gated channels for ions and alter the membrane potential to excite or inhibit cell activity. Most metabotropic receptors are G protein-coupled receptors (GPCRs), which rely on second messengers inside the cell to modulate ion channels or trigger signaling cascades to release calcium from cells (9).

Functional GPCRs can be divided into four families depending on the α subunit type: the G_αs_ family, G_αi/Gαo_ family, G_αq/11_ family, and G_α12/Gα13_ family. The G_αs_ and G_αi/o_ pathways target the cyclic adenosine monophosphate (cAMP)-generating enzyme adenylyl cyclase (AC) to stimulate or inhibit the conversion of cytosolic ATP to cAMP. Cytosolic cAMP determines the activities of ion channels and is considered a second messenger of GPCRs. The effector of the G_q/11_ pathway is phospholipase C-β, which produces the second messengers inositol (1,4,5) trisphosphate (IP3) and diacylglycerol (DAG) to increase cytosolic Ca^2+^ levels.

The pancreatic islets are key parts of glucose homeostasis. Appropriate communication between islets and other organs is needed to maintain glucose homeostasis under different situations (4).

The neuronal populations in the arcuate nucleus of the hypothalamus are crucial for the regulation of energy balance, primarily in the control of food intake. In addition, the normal glucose ranges are set by the biological defended level of glycemia (BDL_G_) in the central nervous system (CNS) (1). Nutrients in circulation, such as glucose, fatty acids and amino acids, can act on islets directly or indirectly to modulate the secretion of hormones from islets to regulate glucose levels. The endocrine cells in islets (α, β and δ cells) share the same vesicular formation and secretion mechanisms as those in the CNS.

In this paper, we will review the function of neurotransmitters and their related receptors in islets and their roles in the development of type 2 diabetes (T2D), and discuss their potential in the treatment of T2D and other metabolic diseases.

## Amino Acids

### Glutamate

Glutamate is a nonessential amino acid in the body and a primary excitatory neurotransmitter in the CNS (10). Glutamate is synthesized in most tissues and is contained in many foods. Intracellular glutamate is formed in mitochondria by glutamate dehydrogenase (GDH) or in the cytosol with the malate-aspartate shuttle (MA). In islets, glutamate mainly comes from α cells, and glutamate infiltration from blood through vessels is insignificant (11). Once formed, intracellular glutamate can be loaded into secretory granules by vesicular glutamate transporter 2 (VGLUT2) and released with glucagon under low-glucose conditions (12).

The glutamate receptors include ionotropic receptors N-methyl-D-aspartate (NMDA) receptors and non-NMDA receptors and metabotropic receptors named mGluR1-8. NMDA receptors can be activated by glycine and glutamate, regulating intracellular sodium and calcium balance. Non-NMDA receptors, including α-amino-3-hydroxy-5-methyl-4-isoxazole propionate (AMPA) receptors and kainate receptors, mediate fast excitatory synaptic transmission. In islets, NMDA receptors are expressed on β cells, and non-NMDA or AMPA/kainate receptors are expressed on α and δ cells. The activation of NMDA receptors on β cells facilitates calcium influx and induces transient insulin secretion. However, the net effect of NMDA receptor activation is inhibiting repolarization after depolarization, resulting in inhibited glucose-stimulated insulin secretion (GSIS). Continuous NMDA receptor activation causes excitotoxicity and death of neurons in the CNS, so does β cells in islets (13). Inhibition of NMDA receptors can enhance GSIS and increase insulin content in islets to improve glucose tolerance in mice (14, 15). AMPA/kainate receptors have less affinity for glutamate than NMDA receptors, and they mediate the excitatory effect of glutamate on neurons in the CNS and α cells in islets. Glutamine is an amino acid with known glucagonotropic effects. Activation of AMPA/kainite receptors on α cells induces influxes of sodium and calcium, leading to depolarization and secretion of glucagon along with intracellular glutamate. The released glutamate can bind with AMPA/kainate receptors again to form a positive feedback loop for glucagon secretion (15, 16). Rat δ cells express the AMPA receptor, and glutamate induces somatostatin release from δ cells under low-glucose conditions, inhibits the secretion of glutamate and glucagon from α cells and forms negative feedback in islets (17).

The mGluRs and related GPCRs have also been shown to regulate islet function, in addition to their roles in the CNS and diabetic neuropathy (18). Group I mGluRs (mGluR1,5) belong to the G_q/11_ family, and group II mGluRs (mGluR2,3) and group III mGluRs (mGluR4,6,7,8) belong to the G_i/o_ and G_s_ families, respectively. The mGluR4 was identified in rat islets and plays a role in PP cells as well as α cells (19, 20). The mGluR8 was detected in male Wistar rat islets, rodent insulin-secreted cell lines RINm5F and MIN6 cells (20), in which the specific agonist of group III receptors inhibited insulin release. However, mGluR8 is also present in α cells of female Sprague–Dawley rats, and the mGluR8 agonist inhibited glucagon release; the group III receptor antagonist reduced this effect (21). The mGluR3 and mGluR5 were detected in rat and human islets (20), and agonists specific to group I or group II increased the release of insulin. The mGluR5 was shown to functionally interact with NMDARs and is needed for optimal insulin secretion (22). The expression of mGluRs in other cells of islets and their function needs further research.

Plasma glutamate levels are elevated in many chronic oxidative stress conditions, such as obesity, insulin resistance, diabetes and cancer (23), and in acute injuries, such as head trauma or cerebral ischemia, creating excitotoxicity and facilitating inflammation (24). Prolonged high glutamate levels accelerate the onset of T2D and increase the risks of cardiovascular diseases in obesity and T2D patients (25, 26). Type 1 diabetes (T1D) patients have higher glutamate levels in their brains, which can be used as an early marker of diabetes-related neurodegenerative diseases (27). Recently, therapies targeting glutamate receptors have been developed to treat T2D. NMDAR antagonists, such as dextromethorphan (DXM), amantadine and memantine, have been successfully used in the treatment of many diseases for decades, including nonproductive cough, nonketotic hyperglycemia, Parkinson’s disease, and Alzheimer’s disease (28). DXM was proven to improve insulin secretion and glycemic control in T2D patients (14, 29). The DXM derivative Lam39M increased the duration and frequency of Ca^2+^ oscillations, extended the time of insulin secretion, and protected mouse and human pancreatic islets from cell death. Lam39M also has lower penetration to the BBB, minimizing the NMDA inhibition effects on the CNS (30). Hence, developing NMDA receptor antagonists with higher specificity to β cells or islets can be a promising road for the treatment of T2D.

### Gamma-Aminobutyric Acid

Gamma-aminobutyric acid (GABA) is synthesized by glutamate and glutamate decarboxylase (GAD) in GABAergic neurons and is the most important inhibitory neurotransmitter in the mammalian CNS. *Bacteroidetes* in the gut are the main source of GABA in the periphery (31). Although the BBB separates peripheral GABA from the CNS, supplemental GABA or precursors of GABA can orally feedback to the CNS through the enteric nervous system (ENS) (32) and GABA receptors on adrenergic and cholinergic nerves (33, 34).

The islet has a density of GABA and GABA receptors comparable to that of the CNS (35). The receptors of GABA include the ligand-gated ion channel GABA_A_ and the GPCR member GABA_B_. GABA_A_ activation induces chloride influx, inhibiting depolarization and reducing excitability of target cells. GABA_B_ couples with the G_i/o_ protein and inhibits cAMP production to exert an inhibitory effect.

The GABA in islets is supplied by β cells by the time of insulin secretion (36). The simultaneously released GABA can bind with GABA_A_ receptors on β cells to inhibit insulin secretion as an autocrine signal. Activation of GABA_A_ receptors also enhanced proliferation of β cells (37). Meanwhile, GABA from β cells can inhibit glucagon secretion and cell proliferation by binding with GABA_A_ receptors on α cells as a paracrine signal (38).When the glucose level is not high enough to evoke action potentials on membranes of β cells, the chloride potential made by the GABA_A_ receptor can moderately depolarize β cells to induce insulin and GABA release (39), but when the glucose level is higher than BDL_G_, GABA will inhibit excessive release of insulin (40). It has been reported that human δ cells express GABA_A_ receptors and that the GABA_A_ antagonist SR95531 reduces the secretion of somatostatin at different glucose concentrations (39).

The mRNAs of GABA_B_ receptors 1 and 2 were identified in islets of rats and human and MIN6 cells, and the GABA_B_ receptor agonist inhibited the release of insulin in the presence of 25 mmol/l glucose (20, 41). Knockout of the GABA_B_ receptor improved glucose tolerance and increased insulin content in the islets of mice, but constitutive absence of the GABA_B_ receptor induced insulin resistance in mice (42).

The GABA content in islets of T2D patients and animals is lower than normal, accompanied by β cell dysfunction and lower insulin content (43). The presence of GAD autoantibodies is important for the diagnosis of autoimmune diabetes (44). In addition to endocrine cells in islets, GABA also works on innate immune cells such as T cells, inhibiting NF-κB signaling and protecting β cells from inflammation, especially in conditions such as T1D and islet transplantations (45).

### Glycine

Glycine is the simplest stable amino acid acting as an inhibitory neurotransmitter in the CNS (46). The glycine level in human cerebrospinal fluid is 5 µM but increases to 150-400 µM in blood. Extracellular glycine can bind with glycine receptors (GlyRs) or be transported into cells by glycine transporters (GlyTs). GlyR is an ionotropic receptor that mediates the transport of chloride. Both GlyR and GlyT are expressed on α cells and β cells of human islets but are barely detected in rodents (47, 48). Although glycine inhibits the activity of neurons in the CNS, it actually excites β cells in islets due to different intracellular chloride concentrations (7 mM in neurons and 32 mM in β cells). Glycine induced depolarization and increased intracellular calcium in β cells (47). Glycine can be coreleased with insulin, and insulin can enhance the effect of glycine-activated current (47, 49). In α cells of islets, glycine can induce glucagon secretion *in vitro* and *in vivo* without a significant change in insulin levels (48, 50). Moreover, glycine can bind with and saturate NMDA receptors as an endogenous antagonist (51), block the excitotoxic effect of glutamate and help maintain normal GSIS (14, 15).

The lower glycine levels in circulation are related to obesity, diabetes and nonalcoholic fatty liver disease (NAFLD), and GlyR expression and glycine-induced currents on β cells of T2D patients is also reduced (52, 53). Supplementation with glycine can alleviate oxidative stress, lower blood pressure, and reduce risks for T2D (54). The elevation of glycine might be protective for people with higher metabolic risks.

### D-Amino Acids

Natural amino acids can be divided into L-type and D-type based on their chirality (except glycine). D-amino acids can be derived from L-amino acids by racemases or under oxidative stress, and some of them also come from food and gut microbiota (55). D-aspartate (D-Asp) and D-serine (D-Ser) are major D-amino acids in mammals.

D-Asp is a racemase product of L-aspartate and a precursor of NMDA and is located at the pineal gland and pituitary in the CNS and adrenal gland (56). D-Asp can stimulate hormone secretion from pituitary glands and the hypothalamus (57). Hyperglycemia can induce the release of D-Asp from the retina of diabetic rats and is related to diabetic retinopathy (58). Although D-Asp is found in α cells and can be released from the rat insulinoma cell line INS-1 along with insulin (59, 60), the function of D-Asp in islets is unclear.

D-Ser is a neuromodulator derived from serine under serine racemase (SRR) in the CNS. D-Ser can inhibit high-fat diet consumption and reduce body weight in mice (61). The content of D-Ser is highest in the CNS, others are also present in the liver, kidney and pancreas (62).The key enzyme of D-Ser synthesis, SRR, is expressed in β cells of human and mouse (63). SRR-knockout mice (Srr-KO) have similar D-Ser content but less insulin content in the pancreas than wild-type (WT) mice (64). Srr-KO mice have lower blood glucose and fasting insulin levels and better glucose tolerance and insulin sensitivity (65). D-Ser acts as a coactivator of NMDA receptors in the CNS, and deletion of SRR in the brain impairs the function of NMDA receptors. Not surprisingly, Srr-KO mice have fewer NMDA receptors on islets, and the NMDA receptor antagonist MK-801 failed to suppress insulin secretion in the islets of Srr-KO mice (65).

A high D-Ser diet increases D-Ser levels in blood and leads to hyperglycemia and impaired glucose tolerance in mice, which can be blocked by α2-adrenergic receptor antagonists (61). Therefore, the adrenergic system might participate in the effect of D-Ser in islets. Polymorphisms of the SRR gene are associated with T2D susceptibility (66) and metformin efficiency (67). Thus researches on the metabolism and effects of D-amino acids are worthwhile in the treatment of T2D.

## Monoamine

Monoamine neurotransmitters (MNTs), including serotonin, norepinephrine, dopamine and histamine, exist broadly in the central and peripheral neural system (68). MNTs are degraded by monoamine oxidase (MAO) or reuptake by monoamine transporters (vesicular monoamine transporters, dopamine transporters, norepinephrine transporters) to halt the effects (69). Dysregulation of MNTs is a primary cause of mental diseases.

### Serotonin

Tryptophan is processed by tryptophan hydroxylase (TPH) and aromatic-L-amino acid decarboxylase (AADC) to form 5-hydroxy tryptamine (5-HT, serotonin) in the CNS and intestines. Entrochromaffin cells (ECCs) contribute approximately 90% of 5-HT in the body, and the others function separately due to the existence of the BBB (70). The receptors of 5-HT belong to GPCRs except 5-HT receptor 3 (5-HT3R), which is a ligand-gated ion channel.

β cells have the key enzymes TPH and ADCC to synthesize 5-HT (71) and vesicular monoamine transporters (VMATs) to load 5-HT on vesicles, so the 5-HT can be released with insulin, GABA and glycine from β cells (72). 5-HT has multiple effects on β cells. Action through 5-HT2BR increases β cell proliferation, and activation of 5-HT3R increases insulin secretion and improves glucose sensitivity. The add-on effects of receptors are critical for compensatory insulin secretion during metabolic stress conditions such as pregnancy and a high-fat diet, by which the expression of TPH also increases. After the physical stresses finish, 5-HT1DR can help to recover β cell mass back to normal (73, 74). In addition to receptor-mediated effects, 5-HT can regulate insulin secretion by serotonylation of GTPase in β cells and facilitate the secretion of insulin (75).

Some 5-HTRs couple with G_i/o_ to reduce cAMP levels and inhibit depolarization. In islets, the inhibitory receptors 5-HT1FR and 5-HT5AR are expressed on α cells, and 5-HT1DR is expressed on δ cells. Therefore, 5-HT inhibits the release of glucagon and SS (76, 77). Although δ cells can also synthesize and release 5-HT, the major source of 5-HT in islets is still β cells, and the inhibition of glucagon by 5-HT also comes from paracrine signals of β cells (78).

5-HT is necessary to maintain glucose homeostasis in humans and mice. The 5-HT1FR on α cells is lower in T2D patients, which might contribute to hyperglycemia (78, 79). Obesity and hyperglycemia are common side effects of the antipsychotic drugs 5-HT receptor antagonists and MAO antagonists, which can be relieved after drug withdrawal (80).

### Catecholamine

Catecholamine (CA) is a group of chemicals synthesized from phenylalanine or tyrosine in the central and peripheral nervous systems, including dopamine, epinephrine and norepinephrine.

Dopamine comes mainly from dopaminergic neurons in the substantia nigra and ventral tegmental area in the CNS and peripheral nerves, the adrenal medulla and some neuroendocrine cells are the main sources of dopamine in the periphery (81). Norepinephrine is synthesized and released by the locus coeruleus in the CNS and sympathetic nerves in the periphery. Epinephrine is released from the adrenal gland and some neurons in the brain stem. Both α and β cells in islets of humans and rodents possess enzymes of CA synthesis and specific transporters for CAs, which means they could be possible sources of CAs in islets (82).

The receptors of CAs are GPCRs. The CAs couple with G_s_ or G_i/o_ subunits to induce excitation and inhibition, respectively. The excitatory receptors increasing cAMP concentration include the D1 and D5 dopamine receptors and the α1, β1, β2 and β3 adrenergic receptors. The inhibitory receptors D2, D3 and D4 dopamine receptors, the α2 adrenergic receptors, reduce the cAMP level in cells (83).

The dopamine receptors D1, D5 (82, 84) and the adrenergic receptor α2 are expressed on β cells (85), while the dopamine receptors D2 and D3 (82) and the adrenergic receptors α1, β1, and β2 are expressed on α cells (86, 87). The effects of dopamine and norepinephrine on islets are inhibiting insulin secretion and promoting glucagon secretion (87, 88). The activation of α2 adrenergic receptors on β cells can suppress insulin secretion, insulin gene expression and insulin synthesis (89, 90). There is clear evidence that overexpression of α2 adrenergic receptors in rodent β cells causes impaired insulin secretion and is associated with spontaneous onset of T2D in GK rats and increased risk of T2D in human (91, 92). Knockout of the α2 adrenergic receptor in mice showed lower blood glucose levels and higher plasma insulin levels, as well as improved glucose tolerance, than the wild type (93). A high level of dopamine inhibits β cell proliferation and induces apoptosis of β cells (94). However, dopamine is necessary for the survival and development of islets, and mice lacking synthesis enzymes or receptors of dopamine develop glucose intolerance and impaired GSIS early in their life (95). β3 adrenergic receptors exist on adipose tissue and induce lipolysis and fatty acid production upon activation (96).

The dopamine transporter (DAT) is located on the surface of β cells. DAT can take up dopamine and store them in vesicles together with intracellularly synthesized dopamine with the help of VMAT2. When β cells depolarize and secrete insulin, dopamine will act on dopamine receptors and adrenergic receptors to inhibit insulin release as an autocrine signal in negative feedback (97).

Bromocriptine is a dopamine D2 receptor agonist used in the treatment of Parkinson’s disease and hyperprolactinemia. Additionally, it has been used as a central antidiabetic drug for years and is still recommended by the American Diabetes Association (ADA) in the latest guidelines (98). Bromocriptine can cause metabolic alterations in patients with insulin resistance and obesity by resetting the hypothalamic circadian rhythm of monoamine neuronal activities. The agonistic action of dopamine may reduce the hypothalamus drive for increased lipid and hepatic glucose production and insulin resistance (99). Elevated prolactin levels are frequently associated with weight gain and obesity, which are common in hyperprolactinemia patients (100). Bromocriptine has the ability to suppress prolactin levels, thereby augmenting glucose tolerance and regulating GSIS (99).

The adrenergic nerve system is activated under stresses such as cold, nervousness and hypoglycemia, promoting heat generation, glucose supplementation, lipolysis and glycolysis. The α cell is able to synthesize and release dopamine and norepinephrine and stimulate glucagon secretion by binding with the adrenergic β1 receptors themselves (82). The autocrine, paracrine and nerve-derived signals in islets can partly explain the higher risk of T2D under prolonged stresses.

T2D patients have higher norepinephrine levels in blood, which inhibits the secretion of insulin (101) and impairs the responsiveness of β cells to adrenergic signals (102). Insulin resistance is characterized by a higher insulin level in the periphery, which will inhibit the reuptake of CAs and activate adrenergic receptors on α and β cells constantly, resulting in damage to glucose homeostasis and accelerating the progression to T2D (79, 103).

Pheochromocytoma is a rare neuroendocrine tumor capable of producing CAs. Hyperglycemia is a common metabolic dysfunction of Pheochromocytoma patients, mostly because of impaired insulin secretion and insulin sensitivity. Epinephrine and norepinephrine disturb glucose homeostasis in different ways. Epinephrin tends to impair insulin secretion, while norepinephrine tends to promote insulin resistance (104). The metabolic mechanism of hyperglycemia in Pheochromocytoma patients depends on the dominant CAs released by Pheochromocytoma and the distribution of adrenergic receptors (105). In addition, it has been reported that the Pheochromocytoma cell line PC12 is rich in D-Asp (62), which can inhibit GSIS by acting on NMDA receptors on β cells (14, 15).

### Histamine

Histamine is synthesized from histidine by histidine decarboxylase (HDC) and stored in mast cells and basophils in an inactive form. The tuberomammillary nucleus (TMN) neurons expressing HDC are the primary source of histamine in the CNS, and the parietal cells in the stomach also express HDC and secrete histamine. mast cell-derived histamine is scarce in the CNS and periphery under normal conditions (106).

Histamine receptors (HRs), including H1-4, are GPCRs with potential ion ligand-gated channel activity. H1R couples with the G_q/11_ subunit to induce calcium current and cell excitation. H2R and H3R are inhibitory receptors that bind the G_s_ and G_i/o_ subunits, respectively. HDC is expressed in α and β cells in islets, but is more highly expressed in tumors (107). The main sources of histamine in islets are mast cells and basophils.

H1R, H2R and H3R are expressed in islet β cells of human and rodents (88, 108). The general effect of histamine on β cells is inhibition of insulin secretion and cell proliferation (109). Agonists of H1R can facilitate insulin secretion and partially counteract cytokine-induced β cell destruction (108, 110). The first generation of the antihistamine drug trimeprazine can incompletely activate H1R and increase β cell proliferation in mice (111). The H1R antagonist cetirizine did not affect diabetes development in NOD mice but did improve the glucose tolerance of high-fat diet-fed mice (112). A selective H3R inverse agonist (antagonist), JNJ-5207852, facilitates insulin secretion and possibly promotes β cell proliferation, while a selective H3R agonist inhibits insulin secretion and cell proliferation of MIN6 cells (108). Both agonists and antagonists of H2R can inhibit insulin secretion, possibly due to species variation and different receptor distributions (88, 108). H3R is expressed on α cells in islets and can inhibit glucagon release (113). Although direct evidence of H4R expression in islets is lacking, blocking H4R with its selective antagonist JNJ-39758979 was efficient in the prevention of diabetic nephropathy progression (114), possibly by inhibiting inflammatory activities in tissues.

Chronic H3R agonist treatment shows multiple metabolic benefits in mice with diet-induced obesity (DIO), such as reducing food intake and body weight and alleviating hyperleptinemia and hyperinsulinemia (115). Proxyfan is an H3R protean agonist that can be used as an agonist, inverse agonist and antagonist of H3R. In T2D mice, oral administration of Proxyfan can lower blood glucose and glycosylated hemoglobin A1c (HbA1c), and intracerebroventricular administration of Proxyfan can increase plasma insulin levels *via* a glucose-independent mechanism (116).

Systemic histaminergic activity is elevated in T2D patients and animals (117). Reactive oxygen species (ROS) are necessary for histamine release, and hyperglycemia can increase ROS and might contribute to the higher histamine levels in T2D patients (118). Higher histamine levels in the plasma of T2D patients also accelerate vascular injury, especially in the aorta, increasing the risk of cardiovascular diseases (119). The islets of T1D patients and streptozotocin-induced diabetic animals have significant mast cell infiltration, and the histamine released by mast cells can aggravate immune injury and lead to cell death through the caspase pathway (120). H2R antagonists are widely used as antacid agents to treat peptic ulcers, and H2R antagonist treatment is associated with a lower prevalence of NAFLD in men (121). It is possible that they have antioxidant properties and direct effects on inflammatory cells, including monocytes, which might prevent inflammation. However, prolonged treatment with H2R antagonists increases the risk of T2D in peptic ulcer patients (122, 123).

## Acetylcholine

Acetylcholine (ACh) is a product of choline and acetyl-coenzyme A (CoA), synthesized by choline acetyltransferase (ChAT) and stored by vesicular acetylcholine transporter (vAChT) in the CNS cholinergic neurons and peripheral autonomic nervous system (sympathetic and parasympathetic nerves). The ACh in islets mainly comes from cholinergic nerve terminals in rodents but comes from both nerves and α cells in humans. The ACh in α cells is loaded into different vesicles from glucagon by vAChT so that they can be released separately (124, 125). The α cells are scattered in human islets but are located on the boundary of islets in rodents, which facilitates paracrine ACh signals from α cells passing to neighboring cells easily in human islets.

Cholinergic receptors can be divided into muscarinic (M) and nicotine (N) receptors. M-type receptors (mAChRs) are GPCRs that couple with the G_q/11_ (M1, M3, M5) or G_i/o_ (M2, M4) subunits to induce excitatory or inhibitory effects on cells. The N-type receptors (nAChRs) are a ligand-gated ion channel consisting of subunits.

The M3 and M5 receptors are present on β cells and can increase insulin release upon activation (124). M3 receptor-deficient mice displayed lower glucagon and insulin levels and impaired postprandial insulin release (126). Decreased expression of M3 receptors in islets was observed under hyperglycemic conditions both *in vivo* and *in vitro* (127). These results suggest the therapeutic potential of the M3 receptor. In addition, ACh can also be involved in paracrine regulation within islets indirectly, which increases or reduces the secretion of somatostatin by binding with M1 receptors on δ cells of human or (128) with M3 and M4 receptors on δ cells of mice (129).The effects mediated by nAChRs are much more complex than those mediated by nAChRs. Isoforms of nAChRs, including α2, α3, α4, α5, α7, β2, and β4 subunits, assemble to form functional nAChRs. The predominant subunits expressed in islets are α5 and β2 in human, α7 and β2 in rodents (130, 131). Nicotine is a natural agonist of nAChRs. Smoking is one of the most famous risk factors for T2D (132). However, chronically treating db/db mice with a small amount of nicotine could improve glucose metabolism and insulin sensitivity (133). The activation of nAChRs can increase β cell mass and enhance insulin secretion (134, 135), as well as protect β cells against cytokine toxicity (130). Loss of α5nAChR in mice was related to increased nicotine intake, of which the impact on glucose homeostasis remains unclear (136). Deletion of α7-nAChR in mice impairs glucose tolerance and causes insulin resistance (133). PUN-282987 is a selective α7-nAChR agonist capable of enhancing insulin sensitivity in muscle, liver and adipose tissue in mice and reducing inflammation *via* the STAT3 pathway (137). Recently, nicotine was found to act on TCF7L2 in the pineal gland and transmit nicotine signals to islets, leading to dysregulation of insulin and glucagon (138).

## Peptides

Peptide signals play a role in both endocrine and neural systems. The term “neuropeptide” is defined by small proteinaceous substances produced and released by neurons through the regulated secretory route and acting on neural substrates. Neuropeptides may act as neurotransmitters or neuromodulators and commonly bind to GPCRs to affect the activities of neurons and other tissues, including pancreatic islets (139, 140). With the help of genetics and multiomics techniques, the family of neuropeptides has expanded quickly during decades. We only discussed several members of the large family and their roles in pancreatic islets due to limited space, including glucagon-like peptide-1 (GLP-1), glucose-dependent insulinotropic polypeptide (GIP), cholecystokinin, oxytocin and vasopressin. Other neuropeptides present in islets, such as peptide tyrosine-tyrosine (PYY) (141), neuropeptide Y (142) and somatostatin (129, 143), have been extensively reviewed elsewhere.

### Incretin

Incretins are a group of metabolic hormones released after eating that augment the secretion of insulin by a blood glucose-dependent mechanism. GLP-1 and GIP are two main candidate molecules that fulfill the criteria for incretin (144). Both GLP-1 and GIP are rapidly inactivated by dipeptideyl peptidase-4 (DPP-4).

GLP-1 is secreted by preproglucagon neurons in the solitary nucleus in the CNS, regulating the activities of the hypothalamus and brain areas (145). GLP-1 in the periphery mostly comes from enteroendocrine L cells (146). The α cells in islets also express the preproglucagon gene and synthesize and secrete propglucagon-derived peptides, including glucagon and GLP-1 (147). The receptors of glucagon and GLP-1 share significant homology and both belong to the G_s_ family of GPCRs and are expressed on β cells in islets, which contribute to the cross-reactivity of glucagon and GLP-1 and induce insulin secretion (148, 149). GLP-1 promotes insulin synthesis and secretion upon stimulation (150) and acts synergistically with glucose to promote insulin gene transcription, mRNA stability, facilitate insulin biosynthesis to replenish insulin stores and prevent exhaustion and apoptosis of β cells (151). GLP-1R agonists enhance β cell proliferation and expand β cell mass even in normoglycemic rodents (152). Some researchers also found GLP-1 receptors on δ cells to control the release of somatostatin (153).

GIP is synthesized by K cells of the duodenum and small intestine. The GIP receptor is a G_s_ family member of GPCRs widely expressed within the CNS (154), α cells and β cells in islets (155) and white adipose tissues (156). The GIP receptor (GIPR) is expressed in similar amounts in α and β cells. GIP stimulates glucagon secretion directly through GIPRs on β cells, while GIP can potentiate the release of glucagon by activating GIPRs on α cells. Glucagon then binds with GLP-1R or the glucagon receptor and stimulates insulin secretion (155, 157). In addition to stimulating the secretion of insulin, such as GLP-1, GIP promotes triglyceride storage by directly activating GIP receptors on adipocytes and indirectly through the lipogenic actions of insulin (158) and enhances glucose uptake and insulin sensitivity of adipocytes, therefore improving the long-term storage of lipids by facilitating the healthy expansion of white adipose tissues (159). Our previous work found that GLP-1 levels in the plasma of T2D and prediabetes patients were lower than those in healthy people but did not differ between T2D and prediabetes patients (160). The genes encoding the human GLP-1 or GIP receptors have not been linked to enhanced genetic susceptibility to diabetes (63). GLP-1-targeted treatments can reduce body weight and risks of cardiovascular events, which are independent of glucose-lowering effects (161, 162). The new generation of anti-diabetic drugs GLP-1 receptor agonists (GLP-1RAs) have shown promising benefits on T2D treatment, as well as the DPP-4 inhibitors, which raises levels of GLP-1 and GIP to augment insulin release after a meal. Central and peripheral administration of GIP receptor agonists lowers body weight by reducing caloric intake (163, 164).

Clinical trials employing liraglutide (human GLP-1 analogue) or exenatide (exendin-4 derivates, animal GLP-1) in addition to intensified insulin regimes in T1D patients did not demonstrate convincing hypoglycemic benefits or describe potential adverse outcomes such as a higher risk of ketoacidosis. Only body weight and insulin doses were consistently reduced (165, 166). Some GLP-1RAs can improve the metabolism and function of the CNS when administered in the periphery they are strongly recommended by ADA for the treatment of T2D and might play positive roles in the treatment of neurodegenerative diseases (167).

### Cholecystokinin

Cholecystokinin (CCK) is secreted by specialized neurons in the CNS and ENS and by enteroendocrine I cells in the intestine (168). The receptors of CCK belong to GPCRs, including CCK1 (CCKA) receptor and CCK2 (CCKB) receptor. CCK plays roles in inducing anxiety and satiety in the CNS, regulating gastric emptying and distension and gallbladder contraction in the gastrointestinal system. It is also a potent stimulator of pancreatic acinar cells that release digestive enzymes after meals (168).

The CCK1 receptor is colocalized with insulin and glucagon in the islets of pigs, rodents and human (169). Activation of CCK1 receptors initiates the Gs and Gq/11 signaling pathways in islet β cells under high- and low-glucose conditions, respectively (170). The biologically active fragment CCK-8 and agonists of the CCK1 receptor can induce insulin secretion and protect β cells against apoptosis (170, 171). CCK2 receptors were found in α cells and δ cells of islets. The CCK2 receptor is also called the gastrin receptor because another gastrointestinal peptide, gastrin, secreted by G cells in the gastric antrum shares a similar sequence with CCK and can bind to the CCK2 receptor with almost the same affinity and potency (172). Gastrin and CCK induced glucagon secretion from purified human islets, which was blocked by an antagonist of CCK2 (173).

Recently, the gene encoding cholecystokinin (Cck) was found to be expressed and upregulated in islets of obese and insulin-resistant mice. CCK was detected in both α and β cells (174). Overexpression of CCK was able to protect β cells from apoptosis, while loss of CCK resulted in reduced islet size and β cell mass and induced a diabetogenic phenotype in mice (174, 175).

The insulinotropic and protective role of CCK in islets makes it a promising therapeutic target for T2D and obesity. Structural modified CCK analogs (such as glycated CCK8 and (pGlu-Gln)-CCK8) have been proven to suppress appetite and improve glucose tolerance and plasma lipids and reduce lipid accumulation in the pancreas and body weight in obese and diabetic rodents (176). CCK has been thought to be an incretin candidate because it originates from guts and responses to nutrients, but CCK receptor blockade failed to affect postprandial insulin secretion, like GLP-1 and GIP (177). However, CCK has the potential to reduce weight and blood glucose and could be an effective adjunct therapy for T2D (178).

### Oxytocin and Vasopressin

Oxytocin and vasopressin have similar structures and are synthesized in the supraoptic nucleus and paraventricular nucleus of the hypothalamus, respectively. They are stored in the neurohypophysis and released upon stimulation, such as labor and hypertonicity. Peripheral OT also comes from the uterus, placenta, amnion and heart.

The receptors of VP (V1a, V1b, V2) and oxytocin are GPCRs expressed broadly in the CNS and periphery (179). The V1a, V1b and oxytocin receptors belong to the G_q/11_ family and induce excitatory effects. The V1a receptor is expressed on vessels to regulate vasoconstriction, and the V1b receptor in the CNS assist the release of andrenocorticotropic hormone (ACTH). Oxytocin plays an anorexigenic role in the CNS and primarily stimulates uterine contraction and lactation in peripheral. The V2 receptor couples with the G_s_ subunits to control water absorption in the kidney (180).

The pancreas is unable to generate oxytocin or vasopressin but has receptors for them on α and β cells (181, 182). The vasopressin receptor V1b is expressed both on α and β cells and can regulate glucose homeostasis in a glucose-dependent way, which increases insulin during hyperglycemia and increases glucagon during hypoglycemia (183, 184). The structure of oxytocin is similar to vasopressin, oxytocin can also bind with V1b on α cells to d induce glucagon secretion (182). V1b knockout mice present reduced fasting insulin, glucagon and blood glucose along with enhanced insulin sensitivity (185).

Oxytocin can protect β cells from metabolic stress and cytokines, promoting insulin secretion and cell proliferation (186, 187). Infusion of oxytocin can improve GSIS in healthy unpregnant humans (188), induce insulin secretion and improve glucose tolerance in DIO mice (189). In pregnant mice, oxytocin can not only initiate parturition but also increase β cell proliferation and mass (189). Gestational diabetes mellitus (GDM) patients have lower plasma oxytocin levels than healthy pregnant women, and oxytocin antagonists can impair insulin secretion and lead to the development of GDM symptoms in pregnant mice (190). Intracerebroventricular application of nanogram amounts of oxytocin causes a rise in insulin levels but can be blocked by atropine, indicating that cholinergic neurons are involved in the CNS regulation of oxytocin on glucose homeostasis (191).

T2D patients have higher vasopressin but lower oxytocin levels in plasma (185). Chronic vasopressin infusion impairs fasting glucose and glucose tolerance in lean rats, which can be observed earlier in the obese rats (184). Treatment with oxytocin can reduce food intake and body weight in human and alleviate metabolic syndromes by improve insulin resistance (192, 193). However, therapies targeting receptors of vasopressin and oxytocin or themselves for diabetes treatment might be difficult due to safety concerns, especially chronic effects on the function of neurohypophysis.

## Purines

Purines are basic components synthesized biologically as nucleosides in cells, functioning as energy molecules and mediating the purinergic signaling cascade by acting on purinergic receptors in the CNS and periphery (194). Adenosine, ATP and other nucleotides, such as uridine triphosphate (UDP), are ligands of purinergic receptors P1 (adenosine receptor), P2Y and P2X receptors. P1 and P2Y receptors are GPCRs, P2X receptors are ligand-gated ion channels.

The P1 receptors A1, A2A, A2B and A3 are all found in islets. A1 receptor is a G_i/o_ member expressed in α and β cells, inhibiting secretion of glucagon and insulin upon activation (195–197). Knockout of A1 receptor mice had no significant effect on the first phase of insulin secretion, but prolonged and amplified the second phase of insulin, glucagon and somatostatin secretion (195). It is also reported that the expression of A1 receptor in α cells declined during the progression of autoimmune diabetes and contributed to the hyperglucagonemia in prediabetic NOD mice (198). The role of another G_i/o_ member A3 receptor in islets is unclear, possibly involved in the survival of β cells (197). A2A receptor and A2B receptor belong to G_s_ family. Activation of A2B induce insulin release in islets of rodents (199). However, the effect of A2A receptor showed species heterogeneity, which is increasing insulin secretion in mice but suppressing insulin secretion in rat β cell lines upon activation (200, 201). While in the islets of zebrafish, the nonselective adenosine receptor agonist 5’-N-ethylcarboxamidoadenosinne (NECA) was found to increase the proliferation of β cells (202).

The P2Y receptors are highly conserved across species, eight P2Y receptors have been discovered in human and six of them are expressed in the islets of either human or rodents, which are P2Y1, P2Y4, P2Y6, P2Y11, P2Y13 and P2Y14 (196, 203–208). Nucleotides including purines and pyrimidines are ligands of P2Y receptors (209). Activation of P2Y1 and P2Y6 receptors increased insulin secretion in MIN6 cells (203), P2Y6 agonist MRS 2957 induced insulin secretion at high glucose concentrations (16.7mM) (206). P2Y13 antagonist MRS2211 increased the secretion of both insulin and glucagon independent of glucose concentrations (204).

All of the known subunits of P2X receptors (P2X1-7) have been found in islets β cell of human and/or rodent (207, 210–214), the P2X7 was also found in α cells of human and mice (207, 210, 215). Activation of P2X receptors induces insulin secretion from β cells. ATP is the ligand of P2X receptors, which is generated during glucose metabolism within cells and is co-released with insulin from β cells (2). Therefore ATP plays an autocrine or paracrine role *via* P2X receptors in the islets (216). The expression of P2X receptors can be various in different developmental and functional state of the islets. The P2X1 and P2X4 receptors in islets of mice only emerged after birth and progressively upregulated with age (210). The P2X7 receptors in β cells of islets were upregulated in non-diabetic obese human compared with the leans possibly as a compensation, but undetectable in T2D patients (215). It is reasonable to consider P2X7 receptor a promising target for treatment of obesity and T2D (217).

## Summary and Discussion

Signals from the central and peripheral nervous systems act on islets collaboratively to maintain glucose homeostasis, which is critical for life. The isolated islets are unable to survive for a long time without the regulation of transmitters and fail to proliferate and secrete insulin, which is also an obstacle for islet transplantation therapies (218, 219). The dysregulation of signals in the CNS can impair BDL_G_ and push the onset of T2D (4). Obesity and glucose dysregulation are common side effects of antipsychotic drugs, which bring a higher risk of developing T2D (80).

Here, we review the evidence of the neurotransmitters presented out of the CNS and their roles in the glucose homeostasis, especially the transmitters that can be synthesized in the islets and their corresponding receptors expressed on cells in islets (**Supplementary Table 1**). With developments in imaging skills and multiomics methods, our understanding of transmitters in organisms has been expanded largely during the past decades (220, 221). As a matter of course, the transmitters synthesized by islets also function on the CNS as feedback signals, such as 5-HT and insulin (222, 223), but the specific mechanism needs further study.

Signal molecules in the CNS and periphery are promising treatment targets for many diseases. It is known that T2D is a metabolic disease caused by multiple risk factors. Hyperglycemia, which is a marker of T2D, can be found in various physiological and pathological conditions. Islet dysfunction in diabetes also promotes the progression of neurodegeneration (224). The success of GLP-1RAs is a good example of transmitter-targeted therapy (161, 162).

The gut-brain axis is a hot section of researches on glucose homeostasis, especially for a great variety of peripheral transmitters are derived from the intestines, not only endocrine cells but also bacteria capable of producing multiple neurotransmitters in the gut, such as dopamine, norepinephrine and GABA (225), which can act on both the central and peripheral regions (225). It is not surprising that some medicines for the digestive system also influence the metabolic system (122). The germ-free mice display increased turnover rates of dopamine, norepinephrine and serotonin in the brain, which could generally reduce pools in systemic circulation independent of microbial production (although factors influencing that increased turnover rate remain to be determined) (226). It is not surprising that some medicines for the digestive system also influence the metabolic system (122). Supplementation with GABA or glycine or the consumption of natural products such as resveratrol, which can influence GLP-1 and 5-HT levels in the peripheral and brain-gut axes, are beneficial to glucose homeostasis and could lower oxidative stress (32, 54, 227). Except for GLP-1, GABA and 5-HT, the intestine secretes dozens of other hormones that probably interact with the CNS to regulate glucose homeostasis, such as GIP, CCK, ghrelin, and peptide YY. The relationship between intestine-derived hormones and metabolic conditions deserves more attention and should be a promising therapeutic target for the treatment of both metabolic diseases and neural diseases.

Therapies targeting on more than one signaling molecules may bring more benefits. For example, LY3298176, a novel dual GIP and GLP-1 receptor agonist developed for the treatment of T2D, has been proven to improve glucose control and reduce body weight in T2D mice and humans (228) Another dual agonist is GEP44, a weight-loss drug acting on both receptors of GLP-1 and peptide YY, which performs better than each single drug and has less unfavorable gastrointestinal reactions (229). Direct supplementation of GABA with sitagliptin (a DPP-4 inhibitor) in T2D patients can promote β cell proliferation and protect cells against apoptosis (230). Recently, the GLP-1/GIP/Glucagon receptor triagonist SAR441255 has been proven effective in glycemic control and weight loss in humans (231). These findings show great potentials of multitarget therapies in the treatment of T2D and obesity and encourage the development of more multitarget therapies in the future. Remarkably, there is interaction among different neurotransmitters and their receptors, for example, L-cells cosecrete ATP together with GLP-1 and PYY, and ATP acts as an additional signal triggering vagal activation and potentially synergizes with the actions of locally elevated peptide hormone concentrations (232). The cross reactivities among neurotransmitter signals expand their effects, clarifying the detail procedures of their interaction can promote the development of new therapies with higher efficiency and less side effects. In addition to drugs, implanted devices directly acting on nerves also find their way in the field. Implantable vagal nerve stimulators (IVNSs) have been approved by the Food and Drug Administration (FDA) to treat epilepsy and depression, which can manipulate target tissues more precisely than planted in the CNS (233, 234). In obesity and T2D patients, the application of IVNSs has also improve glucose homeostasis (235).

Research on transmitters in the central and peripheral nervous systems can extend our understanding of neurodegenerative diseases, mental diseases and metabolic diseases, assisting in disease prevention and the development of new antipsychotic drugs with higher selectivity and fewer side metabolic effects. New therapies based on the function of neural transmitters and corresponding receptors targeting pancreatic islets or beyond are also promising for the treatment of T2D and other metabolic diseases.

## Author Contributions

XP and ST reviewed the literature and wrote the manuscript. NT guided critical discussion of the topic and reviewed and edited the manuscript. All authors contributed to the article and approved the submitted version.

## Conflict of Interest

NT has worked on clinical trials funded by Huadong Medicine Group Company Limited, and received lecture fees from Novo Nosdik. However, theses interests will not impair justice and scientific nature of this study.

The remaining authors declare that the research was conducted in the absence of any commercial or financial relationships that could be construed as a potential conflict of interest.

## Publisher’s Note

All claims expressed in this article are solely those of the authors and do not necessarily represent those of their affiliated organizations, or those of the publisher, the editors and the reviewers. Any product that may be evaluated in this article, or claim that may be made by its manufacturer, is not guaranteed or endorsed by the publisher.
