# Supplementary material for: Potential Therapeutic Targeting Neurotransmitter Receptors in Diabetes
Source: Front Endocrinol (Lausanne). 2022 May 20;13:884549. doi: 10.3389/fendo.2022.884549 (PMC9163348; doi:10.3389/fendo.2022.884549)
Supplement: Supplementary file 1 [file Table_1.docx]

Supplementary Table 1. Neurotransmitters and neurotransmitter receptors expressed in pancreatic islets.

| Molecule types | Neurotransmitter | | Receptors | Receptor type | Distribution of receptors | Reference |
| --- | --- | --- | --- | --- | --- | --- |
| Amino acid | Glutamate | | mGluR5 | Gq/11 | Human and rat α, β cells | (1) |
|  |  | | mGluR3 | Gi/o | Human and rat α, β cells | (1) |
|  |  | | mGluR4 |  | Rat α cells | (1, 2) |
|  |  | | mGluR8 |  | Rat α, β cells; mouse β cells | (1, 3) |
|  |  | | AMPA/Kainate receptor | ligand-gated ion channels | Human α cells; rat δ cells; mouse α, β cells | (4-6) |
|  |  | | NMDA receptor | ligand-gated ion channels | Human and mouse β cells | (7) |
|  | D-Amino acid | D-Asp | NMDA receptor |  |  |  |
|  |  | D-Ser |  |  |  |  |
|  | GABA | | GABA-A | ligand-gated ion channels | Humanα, β and δ cells; mouse α, β cells | (8-10) |
|  |  | | GABA-B | Gi/o | Human, mouse and rat β cells | (1, 11, 12) |
|  | Glycine | | Gly-R | ligand-gated ion channels | Human α, β cells | (13, 14) |
| Monoamines | 5-HT | | 5-HT2 | Gs | Human α, β, δ cells; mouse and rat β cell | (15, 16) |
|  |  | | 5-HT3 | ligand-gated ion channels | Human, mouse and rat β cell | (17, 18) |
|  |  | | 5-HT1 | Gi/o | Human α, β, δ cells; rat α, δ cells | (16, 19-21) |
|  |  | | 5-HT5A |  | Human and rat α cells | (16, 19) |
|  | Catecholamine | Dopamine | D1 | Gs | Human and mouse β cells | (22-24) |
|  |  |  | D2 | Gi/o | Human and mouse, α, β cells, human delta cells | (22, 24) |
|  |  |  | D3 |  | Human and mouse, α, β cells | (22) |
|  |  |  | D5 | Gs | Human δ cells, rat α cells | (23, 24) |
|  |  | Epinephrine/Norepinephrine | α1 |  | Mouse α cells | (25) |
|  |  |  | α2 | Gi/o | Human and mouse β cells | (22, 26, 27) |
|  |  |  | β1 | Gs | Human and mouse α cells | (22, 25) |
|  |  |  | β2 |  | Mouse α cells | (25) |
|  | Histamine | | H1 | Gq/11 | Human, mouse and rat β cell | (17, 18) |
|  |  | | H2 | Gs | Human, mouse and rat β cell | (17, 18) |
|  |  | | H3 | Gi/o | Human and rat β cells, Mouse α and β cells | (17, 18, 28) |
| Acetylcholine | | | M1 | Gq/11 | Human δ cells, mouse β cells | (29, 30) |
|  | | | M3 |  | Mouse β cells and δ cells | (30-32) |
|  | | | M5 |  | Human β cells | (31) |
|  | | | M2 | Gi/o | Mouse β cells | (30) |
|  | | | M4 |  | Mouse β and δ cells | (30, 32) |
|  | | | α7 | ligand-gated ion channels | Mouse and rat β cells | (33) |
|  | | | α5 |  | Human β cells | (34, 35) |
|  | | | β2 |  | Human, mouse and rat islets | (34, 35) |
|  | | | β4 |  | Human and mouse β cells(36) | (36) |
| Peptides | incretin | GLP-1 | GLP-1R | Gs | Human, mouse and rat β cells | (37) |
|  |  | GIP | GIPR | Gs | Human β cells, Mouse α, β | (38) |
|  | Somatostatin | | SSR | Gi/o | Human, mouse and rat, α, β cells | (39) |
|  | Cholecystokinin | | CCK1/A | Gq/11, Gs | Human, pig, mouse and rat, α, β cells | (40) |
|  |  | | CCK2/B | Gq/11 | Human α and δ cells; pig, mouse and rat δ cells | (41, 42) |
|  | Oxytocin | | OTR | Gq/11 | Human and mouse β cells; rat α, β cells | (43, 44) |
|  | Vasopressin | | V1a | Gq/11 | Human, mouse and rat α cells | (45, 46) |
|  |  | | V1b |  | Human and mouse β cells; rat α cells | (45, 46). |
|  |  | | V2 | Gs | Human β cells; rat α, β cells, | (45, 46) |
| Purines | adenosine | | A1 | Gi/o | Mouse α, β cells | (47, 48) |
|  |  | | A2A | Gs | Mouse α and β cells, rat β cells | (48-50) |
|  |  | | A2B | Gs | Mouse and rat β cells | (50) |
|  |  | | A3 | Gi/o | Mouse β cells | (51) |
|  | nucleotides | ADP | P2Y1 | Gq/11 | Human β cells, mouse α and β cells | (48, 52, 53) |
|  |  | UTP | P2Y4 | Gq/11, Gi/o | Rat α and β cells | (54) |
|  |  | UDP | P2Y6 | Gq/11 | Mouse β cells | (52, 55) |
|  |  | ATP | P2Y11 | Gq/11, Gs | Rat β cells | (56) |
|  |  | ADP | P2Y13 | Gi/o | Mouse β cells | (53) |
|  |  | UDP-Glucose | P2Y14 | Gi/o | Human and rat β cells | (57) |
|  | ATP | | P2X1 | ligand-gated ion channels | Mouse β cells | (58-60) |
|  |  | | P2X2 |  | Mouse and rat β cells | (60, 61) |
|  |  | | P2X3 |  | Human, mouse, rat β cells | (59, 61, 62) |
|  |  | | P2X4 |  | Mouse and rat β cells | (58, 60, 61) |
|  |  | | P2X5 |  | Mouse β cells | (60) |
|  |  | | P2X6 |  | Mouse and rat β cells | (61) |
|  |  | | P2X7 |  | Human α and β cells, mouse α cells, rat β cells | (56, 58, 63) |

Different types of neurotransmitters and their receptors are expressed in pancreatic islets. Neurotransmitter receptors can be either G-protein coupled receptors or ligand-gated channels, acting with corresponding ligands to exert stimulatory or inhibitory effects. Some neurotransmitters can bind with receptors of other neurotransmitters, mostly due to their similarity in structures.The location of some receptors differs among different species, so the same neurotransmitter may exert different effect on different species. The receptors have not been detected in islets are not described in the table.

Abbreviations: mGluR, metabolic glutamate receptor; AMPA, α-amino-3-hydroxy-5-methyl-4-isoxazole propionate; NMDA, N-methyl-D-aspartate; D-Asp, D-Aspartate; D-Ser, D-Serine; GABA, Gamma-aminobutyric acid; Gly-R, glycine receptor; GLP-1, glucagon-like peptide-1; GLP-1R, glucagon-like peptide-1 receptor; GIP, glucose-dependent insulinotropic polypeptide; GIPR, glucose-dependent insulinotropic polypeptide receptor; SSR, somatostatin receptor; OTR, oxytocin receptor; ADP, adenosine diphosphate; UTP, uridine triphosphate; UDP, uridine diphosphate; ATP, adenosine triphosphate.

Reference

1. Brice NL, Varadi A, Ashcroft SJH, Molnar E. Metabotropic glutamate and GABA(B) receptors contribute to the modulation of glucose-stimulated insulin secretion in pancreatic beta cells. Diabetologia. 2002;45(2):242-52.

2. Uehara S, Muroyama A, Echigo N, Morimoto R, Otsuka M, Yatsushiro S, et al. Metabotropic glutamate receptor type 4 is involved in autoinhibitory cascade for glucagon secretion by alpha-cells of islet of Langerhans. Diabetes. 2004;53(4).

3. Tong Q, Ouedraogo R, Kirchgessner AL. Localization and function of group III metabotropic glutamate receptors in rat pancreatic islets. Am J Physiol Endocrinol Metab. 2002;282(6):E1324-E33.

4. Cabrera O, Jacques-Silva MC, Speier S, Yang S-N, Köhler M, Fachado A, et al. Glutamate is a positive autocrine signal for glucagon release. Cell Metab. 2008;7(6):545-54.

5. Muroyama A, Uehara S, Yatsushiro S, Echigo N, Morimoto R, Morita M, et al. A novel variant of ionotropic glutamate receptor regulates somatostatin secretion from delta-cells of islets of Langerhans. Diabetes. 2004;53(7):1743-53.

6. Wu Z-Y, Zhu L-J, Zou N, Bombek LK, Shao C-Y, Wang N, et al. AMPA receptors regulate exocytosis and insulin release in pancreatic β cells. Traffic. 2012;13(8):1124-39.

7. Marquard J, Otter S, Welters A, Stirban A, Fischer A, Eglinger J, et al. Characterization of pancreatic NMDA receptors as possible drug targets for diabetes treatment. Nat Med. 2015;21(4):363-72.

8. Feng AL, Xiang Y-Y, Gui L, Kaltsidis G, Feng Q, Lu W-Y. Paracrine GABA and insulin regulate pancreatic alpha cell proliferation in a mouse model of type 1 diabetes. Diabetologia. 2017;60(6):1033-42.

9. Braun M, Ramracheya R, Bengtsson M, Clark A, Walker JN, Johnson PR, et al. Gamma-aminobutyric acid (GABA) is an autocrine excitatory transmitter in human pancreatic beta-cells. Diabetes. 2010;59(7):1694-701.

10. Untereiner A, Xu J, Bhattacharjee A, Cabrera O, Hu C, Dai FF, et al. γ-aminobutyric acid stimulates β-cell proliferation through the mTORC1/p70S6K pathway, an effect amplified by Ly49, a novel γ-aminobutyric acid type A receptor positive allosteric modulator. Diabetes Obes Metab. 2020;22(11):2021-31.

11. Bonaventura MM, Catalano PN, Chamson-Reig A, Arany E, Hill D, Bettler B, et al. GABAB receptors and glucose homeostasis: evaluation in GABAB receptor knockout mice. Am J Physiol Endocrinol Metab. 2008;294(1):E157-E67.

12. Braun M, Wendt A, Buschard K, Salehi A, Sewing S, Gromada J, et al. GABAB receptor activation inhibits exocytosis in rat pancreatic beta-cells by G-protein-dependent activation of calcineurin. J Physiol. 2004;559(Pt 2):397-409.

13. Li C, Liu C, Nissim I, Chen J, Chen P, Doliba N, et al. Regulation of glucagon secretion in normal and diabetic human islets by γ-hydroxybutyrate and glycine. J Biol Chem. 2013;288(6):3938-51.

14. Yan-Do R, Duong E, Manning Fox JE, Dai X, Suzuki K, Khan S, et al. A Glycine-Insulin Autocrine Feedback Loop Enhances Insulin Secretion From Human β-Cells and Is Impaired in Type 2 Diabetes. Diabetes. 2016;65(8):2311-21.

15. Kim H, Toyofuku Y, Lynn FC, Chak E, Uchida T, Mizukami H, et al. Serotonin regulates pancreatic beta cell mass during pregnancy. Nat Med. 2010;16(7):804-8.

16. Bennet H, Balhuizen A, Medina A, Dekker Nitert M, Ottosson Laakso E, Essén S, et al. Altered serotonin (5-HT) 1D and 2A receptor expression may contribute to defective insulin and glucagon secretion in human type 2 diabetes. Peptides. 2015;71:113-20.

17. Nakamura T, Yoshikawa T, Noguchi N, Sugawara A, Kasajima A, Sasano H, et al. The expression and function of histamine H₃ receptors in pancreatic beta cells. Br J Pharmacol. 2014;171(1):171-85.

18. Nagata M, Yokooji T, Nakai T, Miura Y, Tomita T, Taogoshi T, et al. Blockade of multiple monoamines receptors reduce insulin secretion from pancreatic β-cells. Sci Rep. 2019;9(1):16438.

19. Uvnäs-Moberg K, Ahlenius S, Alster P, Hillegaart V. Effects of selective serotonin and dopamine agonists on plasma levels of glucose, insulin and glucagon in the rat. Neuroendocrinology. 1996;63(3):269-74.

20. Almaça J, Molina J, Menegaz D, Pronin AN, Tamayo A, Slepak V, et al. Human Beta Cells Produce and Release Serotonin to Inhibit Glucagon Secretion from Alpha Cells. Cell Rep. 2016;17(12):3281-91.

21. Gromada J, Chabosseau P, Rutter GA. The α-cell in diabetes mellitus. Nat Rev Endocrinol. 2018;14(12):694-704.

22. Aslanoglou D, Bertera S, Sánchez-Soto M, Benjamin Free R, Lee J, Zong W, et al. Dopamine regulates pancreatic glucagon and insulin secretion via adrenergic and dopaminergic receptors. Transl Psychiatry. 2021;11(1):59.

23. Zhang Y, Zheng R, Meng X, Wang L, Liu L, Gao Y. Pancreatic Endocrine Effects of Dopamine Receptors in Human Islet Cells. Pancreas. 2015;44(6):925-9.

24. Chen Y, Hong F, Chen H, Fan R-F, Zhang X-L, Zhang Y, et al. Distinctive expression and cellular distribution of dopamine receptors in the pancreatic islets of rats. Cell Tissue Res. 2014;357(3):597-606.

25. Vieira E, Liu Y-J, Gylfe E. Involvement of alpha1 and beta-adrenoceptors in adrenaline stimulation of the glucagon-secreting mouse alpha-cell. Naunyn Schmiedebergs Arch Pharmacol. 2004;369(2):179-83.

26. Filipponi P, Gregorio F, Ferrandina C, Nicoletti I, Mannarelli C, Pippi R, et al. Alpha-adrenergic system in the modulation of pancreatic A and B cell function in normal rats. Diabetes Res Clin Pract. 1986;2(6):325-36.

27. Redmon JB, Towle HC, Robertson RP. Regulation of human insulin gene transcription by glucose, epinephrine, and somatostatin. Diabetes. 1994;43(4):546-51.

28. Nakamura T, Yoshikawa T, Naganuma F, Mohsen A, Iida T, Miura Y, et al. Role of histamine H3 receptor in glucagon-secreting αTC1.6 cells. FEBS Open Bio. 2015;5:36-41.

29. Molina J, Rodriguez-Diaz R, Fachado A, Jacques-Silva MC, Berggren P-O, Caicedo A. Control of insulin secretion by cholinergic signaling in the human pancreatic islet. Diabetes. 2014;63(8):2714-26.

30. Miguel JC, Abdel-Wahab YHA, Mathias PCF, Flatt PR. Muscarinic receptor subtypes mediate stimulatory and paradoxical inhibitory effects on an insulin-secreting beta cell line. Biochim Biophys Acta. 2002;1569(1-3):45-50.

31. Rodriguez-Diaz R, Dando R, Jacques-Silva MC, Fachado A, Molina J, Abdulreda MH, et al. Alpha cells secrete acetylcholine as a non-neuronal paracrine signal priming beta cell function in humans. Nat Med. 2011;17(7):888-92.

32. Rorsman P, Huising MO. The somatostatin-secreting pancreatic δ-cell in health and disease. Nat Rev Endocrinol. 2018;14(7):404-14.

33. Gupta D, Lacayo AA, Greene SM, Leahy JL, Jetton TL. β-Cell mass restoration by α7 nicotinic acetylcholine receptor activation. J Biol Chem. 2018;293(52):20295-306.

34. Klee P, Bosco D, Guérardel A, Somm E, Toulotte A, Maechler P, et al. Activation of Nicotinic Acetylcholine Receptors Decreases Apoptosis in Human and Female Murine Pancreatic Islets. Endocrinology. 2016;157(10):3800-8.

35. Somm E, Guérardel A, Maouche K, Toulotte A, Veyrat-Durebex C, Rohner-Jeanrenaud F, et al. Concomitant alpha7 and beta2 nicotinic AChR subunit deficiency leads to impaired energy homeostasis and increased physical activity in mice. Mol Genet Metab. 2014;112(1):64-72.

36. Ganic E, Singh T, Luan C, Fadista J, Johansson JK, Cyphert HA, et al. MafA-Controlled Nicotinic Receptor Expression Is Essential for Insulin Secretion and Is Impaired in Patients with Type 2 Diabetes. Cell Rep. 2016;14(8):1991-2002.

37. Tornehave D, Kristensen P, Rømer J, Knudsen LB, Heller RS. Expression of the GLP-1 receptor in mouse, rat, and human pancreas. J Histochem Cytochem. 2008;56(9):841-51.

38. El K, Gray SM, Capozzi ME, Knuth ER, Jin E, Svendsen B, et al. GIP mediates the incretin effect and glucose tolerance by dual actions on α cells and β cells. Sci Adv. 2021;7(11).

39. Gao R, Yang T, Zhang Q. δ-Cells: The Neighborhood Watch in the Islet Community. Biology (Basel). 2021;10(2).

40. Morisset J, Julien S, Lainé J. Localization of cholecystokinin receptor subtypes in the endocine pancreas. J Histochem Cytochem. 2003;51(11):1501-13.

41. Morisset J, Wong H, Walsh JH, Lainé J, Bourassa J. Pancreatic CCK(B) receptors: their potential roles in somatostatin release and delta-cell proliferation. Am J Physiol Gastrointest Liver Physiol. 2000;279(1):G148-G56.

42. Saillan-Barreau C, Dufresne M, Clerc P, Sanchez D, Corominola H, Moriscot C, et al. Evidence for a functional role of the cholecystokinin-B/gastrin receptor in the human fetal and adult pancreas. Diabetes. 1999;48(10):2015-21.

43. Suzuki M, Honda Y, Li M-Z, Masuko S, Murata Y. The localization of oxytocin receptors in the islets of Langerhans in the rat pancreas. Regul Pept. 2013;183:42-5.

44. Mohan S, Khan D, Moffett RC, Irwin N, Flatt PR. Oxytocin is present in islets and plays a role in beta-cell function and survival. Peptides. 2018;100:260-8.

45. Mohan S, Moffett RC, Thomas KG, Irwin N, Flatt PR. Vasopressin receptors in islets enhance glucose tolerance, pancreatic beta-cell secretory function, proliferation and survival. Biochimie. 2019;158:191-8.

46. Abu-Basha EA, Yibchok-Anun S, Hsu WH. Glucose dependency of arginine vasopressin-induced insulin and glucagon release from the perfused rat pancreas. Metabolism. 2002;51(9):1184-90.

47. Salehi A, Parandeh F, Fredholm BB, Grapengiesser E, Hellman B. Absence of adenosine A1 receptors unmasks pulses of insulin release and prolongs those of glucagon and somatostatin. Life Sci. 2009;85(11-12):470-6.

48. Tudurí E, Filiputti E, Carneiro EM, Quesada I. Inhibition of Ca2+ signaling and glucagon secretion in mouse pancreatic alpha-cells by extracellular ATP and purinergic receptors. Am J Physiol Endocrinol Metab. 2008;294(5):E952-E60.

49. Andersson O, Adams BA, Yoo D, Ellis GC, Gut P, Anderson RM, et al. Adenosine signaling promotes regeneration of pancreatic β cells in vivo. Cell Metab. 2012;15(6):885-94.

50. Hayashi M. Expression of Adenosine Receptors in Rodent Pancreas. Int J Mol Sci. 2019;20(21).

51. Ohtani M, Oka T, Ohura K. Possible involvement of A₂A and A₃ receptors in modulation of insulin secretion and β-cell survival in mouse pancreatic islets. Gen Comp Endocrinol. 2013;187:86-94.

52. Balasubramanian R, Ruiz de Azua I, Wess J, Jacobson KA. Activation of distinct P2Y receptor subtypes stimulates insulin secretion in MIN6 mouse pancreatic beta cells. Biochem Pharmacol. 2010;79(9):1317-26.

53. Amisten S, Meidute-Abaraviciene S, Tan C, Olde B, Lundquist I, Salehi A, et al. ADP mediates inhibition of insulin secretion by activation of P2Y13 receptors in mice. Diabetologia. 2010;53(9):1927-34.

54. Coutinho-Silva R, Parsons M, Robson T, Lincoln J, Burnstock G. P2X and P2Y purinoceptor expression in pancreas from streptozotocin-diabetic rats. Mol Cell Endocrinol. 2003;204(1-2):141-54.

55. Balasubramanian R, Maruoka H, Jayasekara PS, Gao Z-G, Jacobson KA. AMP-activated protein kinase as regulator of P2Y(6) receptor-induced insulin secretion in mouse pancreatic β-cells. Biochem Pharmacol. 2013;85(7):991-8.

56. Lee DH, Park K-S, Kim D-R, Lee J-W, Kong ID. Dual effect of ATP on glucose-induced insulin secretion in HIT-T15 cells. Pancreas. 2008;37(3):302-8.

57. Parandeh F, Amisten S, Verma G, Mohammed Al-Amily I, Dunér P, Salehi A. Inhibitory effect of UDP-glucose on cAMP generation and insulin secretion. J Biol Chem. 2020;295(45):15245-52.

58. Coutinho-Silva R, Parsons M, Robson T, Burnstock G. Changes in expression of P2 receptors in rat and mouse pancreas during development and ageing. Cell Tissue Res. 2001;306(3):373-83.

59. Silva AM, Rodrigues RJ, Tomé AR, Cunha RA, Misler S, Rosário LM, et al. Electrophysiological and immunocytochemical evidence for P2X purinergic receptors in pancreatic beta cells. Pancreas. 2008;36(3):279-83.

60. Ohtani M, Ohura K, Oka T. Involvement of P2X receptors in the regulation of insulin secretion, proliferation and survival in mouse pancreatic β-cells. Cell Physiol Biochem. 2011;28(2):355-66.

61. Richards-Williams C, Contreras JL, Berecek KH, Schwiebert EM. Extracellular ATP and zinc are co-secreted with insulin and activate multiple P2X purinergic receptor channels expressed by islet beta-cells to potentiate insulin secretion. Purinergic Signal. 2008;4(4):393-405.

62. Jacques-Silva MC, Correa-Medina M, Cabrera O, Rodriguez-Diaz R, Makeeva N, Fachado A, et al. ATP-gated P2X3 receptors constitute a positive autocrine signal for insulin release in the human pancreatic beta cell. Proc Natl Acad Sci U S A. 2010;107(14):6465-70.

63. Glas R, Sauter NS, Schulthess FT, Shu L, Oberholzer J, Maedler K. Purinergic P2X7 receptors regulate secretion of interleukin-1 receptor antagonist and beta cell function and survival. Diabetologia. 2009;52(8):1579-88.
